# Peripheral artery disease and all-cause and cardiovascular mortality in patients with NAFLD

**DOI:** 10.1007/s40618-022-01792-9

**Published:** 2022-04-02

**Authors:** S. Ciardullo, E. Bianconi, R. Cannistraci, P. Parmeggiani, E. M. Marone, G. Perseghin

**Affiliations:** 1 Department of Medicine and Rehabilitation, Policlinico di Monza, Via Modigliani 10, 20900 Monza, MB Italy; 2 Department of Medicine and Surgery, University of Milano Bicocca, Università degli Studi di Milano Bicocca, Milan, Italy; 3 Vascular Surgery, Department of Clinical-Surgical, Diagnostic and Pediatric Sciences, University of Pavia, Pavia, Italy; 4 Department of Vascular Surgery, Policlinico di Monza, Monza, Italy

**Keywords:** NAFLD, ABI, Mortality, CVD

## Abstract

**Purpose:**

Cardiovascular disease (CVD) is the first cause of death in patients with non-alcoholic fatty liver disease (NAFLD) and risk stratification is recommended by current guidelines. The aim of this study is to assess the prevalence of peripheral arterial disease (PAD) in patients with NAFLD and its association with all-cause and cardiovascular disease (CVD) mortality.

**Methods:**

9145 participants 40 years or older attended a mobile examination center visit in the 1999–2004 cycles of the National Health and Nutrition Examination Survey. PAD was defined as an ankle-brachial index (ABI) < 0.90 in either of the legs and mortality data through December 2015 were obtained from the National Death Index. NAFLD was defined by a fatty liver index ≥ 60 in the absence of other liver conditions, leading to a final sample of 3094 subjects.

**Results:**

The overall prevalence of PAD was 5.9% (95% CI 5.0–6.9). Over a median follow-up of 13 years, 876 participants died, 208 of cardiovascular causes. Incidence rates of all-cause mortality (for 1000 person-years) were 20.2 (95% CI 18.7–21.7) and 70.0 (95% CI 60.1–81.6) for participants without and with PAD, respectively. Multivariable-adjusted Cox proportional hazard models showed that PAD was associated with a higher risk of all-cause (1.8, 95% CI 1.4–2.4) and cardiovascular mortality (HR 2.5, 95% CI 1.5–4.3) after adjustment for potential confounders including prevalent CVD.

**Conclusion:**

Current guidelines strongly encourage the screening of CVD in patients with NAFLD and the use of the simple and inexpensive measurement of ABI in routine clinical practice may find indication.

**Supplementary Information:**

The online version contains supplementary material available at 10.1007/s40618-022-01792-9.

## Introduction

Fueled by the ongoing obesity epidemic, non-alcoholic fatty liver disease (NAFLD) has become by far the most common chronic liver condition worldwide, affecting a quarter of the general adult population [1, 2]. Its high prevalence made it one of the most common causes of advanced liver fibrosis and cirrhosis in recent years [3], even though the absolute risk of developing these complications is relatively low for the affected individual. Indeed, while conflicting evidence exists on whether NAFLD might be considered an independent risk factor for cardiovascular disease (CVD) [4, 5], CVD prevalence is higher in patients with NAFLD compared with matched controls; moreover, CVD still represents the most common cause of death in these patients [6]. Based on these premises, current guidelines strongly recommend screening of the cardiovascular system in all patients with NAFLD, at least by detailed risk factor assessment [7].

Peripheral arterial disease (PAD), a manifestation of systemic atherosclerosis, has been reported as a powerful predictor of CVD and mortality, regardless of the presence of associated symptoms [8]. Nonetheless, it represents a condition that is often neglected, with fewer than 50% of the affected individuals being aware of their condition [9]. It can be diagnosed in routine clinical practice using a simple and inexpensive test with high sensitivity and specificity, such as the ankle-brachial index (ABI) [10]. While several studies evaluated the prevalence of PAD in the general population [11] or in patients with diabetes, little is known on its frequency and prognostic impact in patients with NAFLD.

In the present population-based cohort study, we assessed the prevalence of PAD, diagnosed based on ABI measurements, in patients with NAFLD that participated in the 1999–2004 cycles of the National Health and Nutrition Examination Survey (NHANES) and evaluated its association with all-cause and cardiovascular mortality.

## Methods

This is an analysis of data from the 1999–2004 cycles of NHANES, which was conducted in the United States by the National Center for Health Statistics. It is an ongoing cross-sectional complex survey aimed at including individuals representative of the general, non-institutionalized population of all ages. To this end, it applies a stratified, multistage, clustered probability sampling design with oversampling of non-Hispanic black, Hispanic and Asian persons, people with low income and older adults. The survey consists of a structured interview conducted in the home, followed by a standardized health examination that includes a physical examination as well as laboratory tests. Full methodology of data collection is available elsewhere [12]. The original survey was approved by the Centers for Disease Control and Prevention Research Ethics Review Board and written informed consent was obtained from all adult participants. The present analysis was deemed exempt by the Institutional Review Board at our institution, as the dataset used in the analysis was completely de-identified.

### Definition of NAFLD and peripheral arterial disease

NAFLD was diagnosed according to a fatty liver index (FLI) value ≥ 60 in the absence of chronic liver disease and significant alcohol consumption. FLI is a simple non-invasive score based on body mass index (BMI), waist circumference, triglycerides and γ-glutamyl-trans-peptidase levels. It was derived in an Italian population using abdominal ultrasound as a comparator [13] and subsequently validated in the US, in which it showed good diagnostic accuracy compared with ^1^H MR spectroscopy (AUC 0.781)[14]. To evaluate the risk of advanced liver fibrosis we calculated the Fibrosis-4 score (FIB-4), which is based on age, aspartate aminotransferase (AST), alanine aminotransferase (ALT) and platelet count as originally proposed [15].

PAD was determined using the ankle-brachial index (ABI), a simple and accurate non-invasive procedure. It was measured in adults aged ≥ 40 years during the examination component of the survey. Systolic blood pressure (SBP) was measured on the right arm and both ankles (posterior tibial artery). The size of the arm cuff used for each participant was based on the arm circumference. The left arm was used in the case the participant had a condition associated with the right arm that would interfere with the measurement. SBP was measured twice at each site in participants aged 40–59 years and once in participants aged ≥ 60 years. ABI was obtained in both legs by dividing the mean SBP in the leg by the mean SBP in the arm. We defined PAD as an ABI < 0.90 in either leg.

### Other variables of interest

Participants self-reported age, sex, race-ethnicity (categorized as non-Hispanic white, non-Hispanic black, Hispanic or other), education, smoking status and previous medical history. Body measurements including height (cm), weight (kg) and waist circumference (cm) were ascertained during the mobile examination center visit; BMI was calculated as weight in kilograms divided by height in meters squared.

Alcohol consumption was estimated based on self-reported data on the amount and frequency of alcohol use within the previous year. It was considered significant if > 1 drink per day for women and > 2 drinks per day for men on average.

Hypertension was defined as an SBP value ≥ 140 mmHg and/or a diastolic blood pressure (DBP) value ≥ 90 mmHg or currently taking antihypertensive drugs [16]. The remaining participants were further categorized as having optimal (SBP < 120 mmHg and Diastolic BP < 80 mmHg), normal (SBP 120–129 mmHg and/or DBP 80–84 mmHg) and high normal BP (SBP 130–139 mmHg and/or DBP 85–89 mmHg). Diabetes was diagnosed if any of the following conditions were met: 1) A self-reported diagnosis of diabetes. 2) Use of anti-diabetic drugs. 3) A Hemoglobin A1c (HbA1c) level ≥ 6.5% (48 mmol/mol). 4) A fasting plasma glucose ≥ 126 mg/dl. 5) A random plasma glucose ≥ 200 mg/dl [17].

Laboratory methods for measurements of HbA1c, glucose, lipid profile, ALT, AST, GGT, platelet count, creatinine, cholesterol and triglycerides levels are reported in detail elsewhere [18]. Hepatitis C virus infection was indicated by the presence of viral RNA and/or a confirmed antibody test and hepatitis B virus infection as a positive surface antigen test, as described [19]. Estimated glomerular filtration rate (eGFR) was computed according to the Chronic Kidney Disease Epidemiology Collaboration (CKD-EPI) equation and CKD was defined as an eGFR < 60 ml/min/1.73 m^2^. Diagnoses of heart failure (HF), coronary artery disease (CAD) and stroke were based on self-report. Patients reporting either CAD or stroke were classified as having cardiovascular disease (CVD).

### All-cause and cardiovascular mortality

Mortality data from death certificates from the National Death Index were linked to NHANES. The present analysis is based on the public-use linked mortality files for NHANES 1999–2004, which include follow-up time and cause of death for adult participants through 31 December 2015. Cardiovascular mortality was defined as death due to heart disease or cerebrovascular disease, as reported on death certificate records. More information on the linkage method and analytic guidelines is available on the specific webpage of the National Center for Health Statistics [20].

### Statistical analysis

All analyses were conducted using Stata version 16 (StataCorp, College Station, TX), accounting for the complex survey design of NHANES. We used appropriate weighting for each analysis, as suggested by the NCHS to obtain estimates that were generalizable to the U.S. population aged 40 years or older. Data are expressed as weighted proportions (Standard Error (SE)) for categorical variables and as weighted means (SE) for continuous variables. Participants’ characteristics by PAD status were compared using linear regression for continuous variables and the design-adjusted Rao–Scott chi-square test for categorical variables.

Kaplan–Meier survival curves for all-cause and cardiovascular mortality were generated stratified by presence of PAD. Cox proportional hazard models were applied to evaluate the association between PAD and all-cause and cardiovascular mortality after adjustment for potential confounders. Covariates included in Model 1 were age, race-ethnicity, education and BMI, while Model 2 adjusted for all variables included in Model 1 plus diabetes, cigarette smoke, blood pressure status and prevalent cardiovascular disease. In sensitivity analyses, the same Models were applied in men and women, separately. A two-tailed value of p < 0.05 was considered statistically significant.

## Results

### Analysis sample

**Fig. 1:**
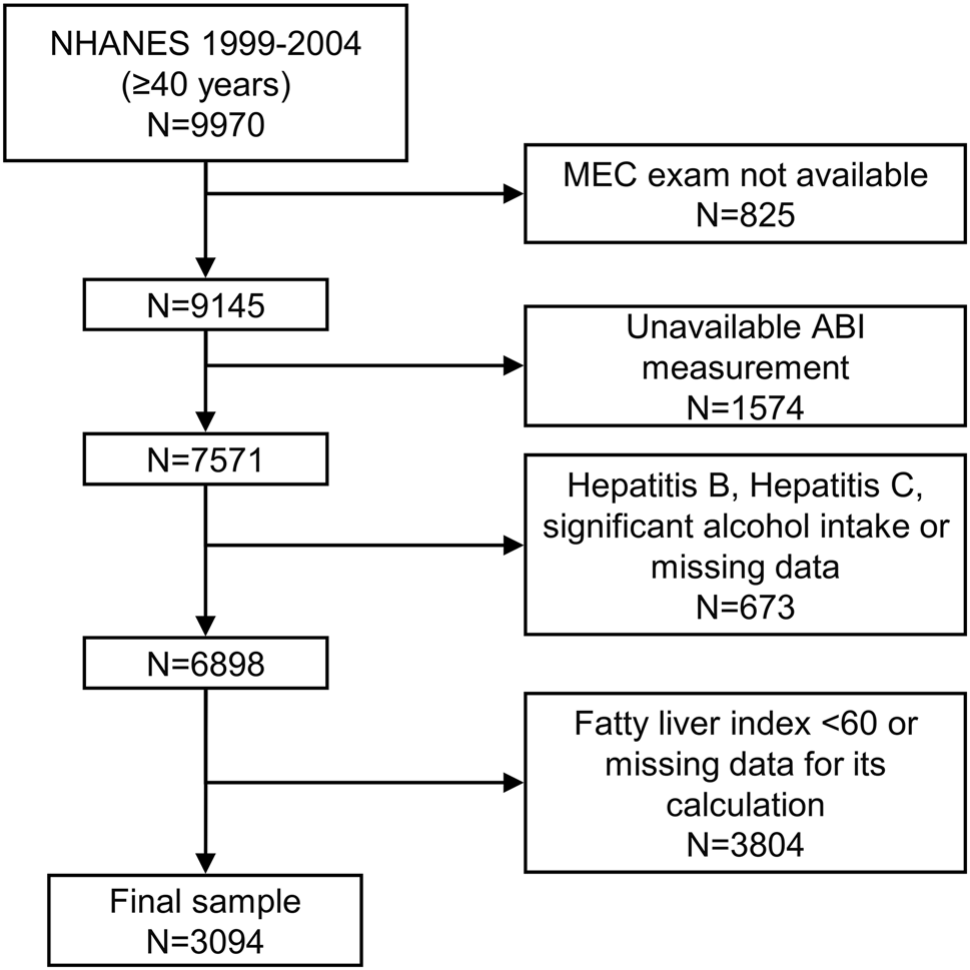
Flow chart of the study participants. *ABI* ankle-brachial index, *NHANES* National Health and Nutrition Examination Survey

9145 participants 40 years or older attended a mobile examination center visit. We initially excluded individuals without an available ABI measurement (*n* = 1574), leading to a population of 7571 participants eligible for PAD assessment. Among these, 673 were excluded for evidence of viral hepatitis, significant alcohol consumption or missing data on these variables. Finally, we excluded 3804 participants with FLI < 60 or missing data for its calculation, leading to a final sample of 3094 participants, as shown in Fig. 1.

### Features of the studied population

The overall weighted prevalence of PAD in US adults with NAFLD aged 40 years or older was 5.9% (95% CI 5.0–6.9). Features of the studied population according to the presence of PAD are shown in Table 1. Participants with PAD were older, more commonly female and of non-Hispanic black ethnicity, while no significant difference was identified in BMI. Adults with PAD were also more frequently former or current cigarette smokers and had a higher prevalence of hypertension and CKD. Prevalent CVD was higher in participants with versus without PAD (35.9% vs. 13.2%), with a similar finding being present for prevalent heart failure. No significant differences were found in lipid profile, while patients with PAD had a higher HbA1c and a higher prevalence of diabetes, as well as a higher prevalence of advanced liver fibrosis according to FIB-4.

**Table 1:** Baseline characteristics of the study population according to ankle-brachial index (ABI)

	ABI ≥ 0.9 (*n* = 2839)	ABI < 0.9 (*n* = 255)	*p*-value
Age (years)	55.7 (0.2)	67.2 (1.0)	< 0.001
Female (%)	42.0 (1.0)	54.8 (4.0)	0.004
Race-Ethnicity (%)			0.007
Non-Hispanic white	77.8 (1.9)	78.9 (2.5)	
Hispanic	10.5 (1.6)	5.9 (2.0)	
Non-Hispanic black	8.6 (1.0)	15.3 (2.4)	
Other	3.1 (0.6)	0.0 (0.0)	
Cigarette smoke (%)			0.007
Never	46.9 (1.6)	35.2 (3.9)	
Past	35.5 (1.3)	46.6 (4.2)	
Current	17.6 (0.8)	18.2 (2.6)	
BMI (kg/m^2^)	32.5 (0.1)	32.0 (0.4)	0.512
SBP (mmHg)	130.0 (0.5)	139.7 (1.9)	< 0.001
DBP (mmHg)	75.7 (0.3)	68.6 (1.2)	< 0.001
Total cholesterol (mg/dL)	217.1 (1.5)	211.0 (3.8)	0.124
Triglycerides (mg/dL)	207.3 (5.2)	213.9 (17.6)	0.711
HDL Cholesterol (mg/dL)	45.6 (0.4)	47.0 (1.2)	0.310
LDL Cholesterol (mg/dL)	132.4 (1.2)	123.0 (3.1)	0.010
eGFR (ml/min/1.73 m^2^)	88.4 (0.7)	73.3 (2.0)	< 0.001
ABI	1.1 (0.0)	0.8 (0.0)	< 0.001
HbA1c (%)	5.8 (0.0)	6.3 (0.1)	0.002
Diabetes (%)	17.8 (0.9)	32.7 (4.0)	< 0.001
Blood pressure category (%)			< 0.001
Optimal	19.0 (1.0)	5.9 (1.7)	
Normal	15.3 (0.8)	11.4 (2.5)	
High-normal	12.9 (0.7)	5.8 (1.7)	
Hypertension	52.7 (1.5)	76.9 (3.1)	
CKD (%)	7.7 (0.5)	28.3 (3.6)	< 0.001
Heart failure (%)	3.2 (0.4)	14.0 (2.5)	< 0.001
CVD (%)	13.2 (0.9)	35.9 (3.9)	< 0.001
Statin use (%)	18.5 (1.0)	34.1 (3.9)	< 0.001
FIB-4 (%)			< 0.001
< 1.3	75.4 (0.9)	49.6 (3.9)	
1.3–2.66	23.1 (0.8)	44.4 (3.6)	
≥ 2.67	1.5 (0.3)	6.0 (2.5)	

Data are expressed as weighted proportions (Standard Error (SE)) for categorical variables and as weighted means (SE) for continuous variables. Linear regression and Rao–Scott chi-square test were used to compare groups

*BMI* Body Mass Index, *CKD* chronic kidney disease, *CVD* cardiovascular disease, *UACR* urinary albumin–creatinine ratio, *HbA1c* Hemoglobin A1c, *HDL* high-density lipoprotein, *SBP* systolic blood pressure, *DBP* diastolic blood pressure, *PAD* peripheral arterial disease

### PAD and mortality

Over a median follow-up of 13 years, 876 participants died in total, 208 of cardiovascular causes. Incidence rates of all-cause mortality (for 1000 person-years) were 20.15 (95% CI 18.72–21.69) and 70.02 (95% CI 60.08–81.60) for participants without and with PAD, respectively. Table 2 summarizes incidence rates for all-cause and cardiovascular mortality in men and women with and without PAD.

**Table 2:** Number of events and incidence rates for all-cause and cardiovascular mortality in US adults by ankle-brachial index (ABI) values

	All-cause mortality		Cardiovascular mortality	
	Events (*n/N*)	Incidence rate per 1000 person-years (95% CI)	Events (*n/N*)	Incidence rate per 1000 person-years (95% CI)
Entire cohort				
No PAD	712/2839	20.15 (18.72–21.69)	163/2839	4.61 (3.95–5.38)
PAD	164/255	70.02 (60.08–81.60)	45/255	19.21 (14.34–25.73)
Men				
No PAD	447/1606	22.80 (20.78–25.01)	116/1606	5.92 (4.93–7.10)
PAD	87/124	81.32 (65.91–100.34)	29/124	27.11 (18.84–39.01)
Women				
No PAD	265/1233	16.85 (14.94–19.01)	47/1233	2.99 (2.24–3.98)
PAD	77/131	60.51 (48.40–75.66)	16/131	12.57 (7.70–20.53)

PAD (peripheral arterial disease) was defined as an ABI < 0.9 in at least one leg

**Fig. 2:**
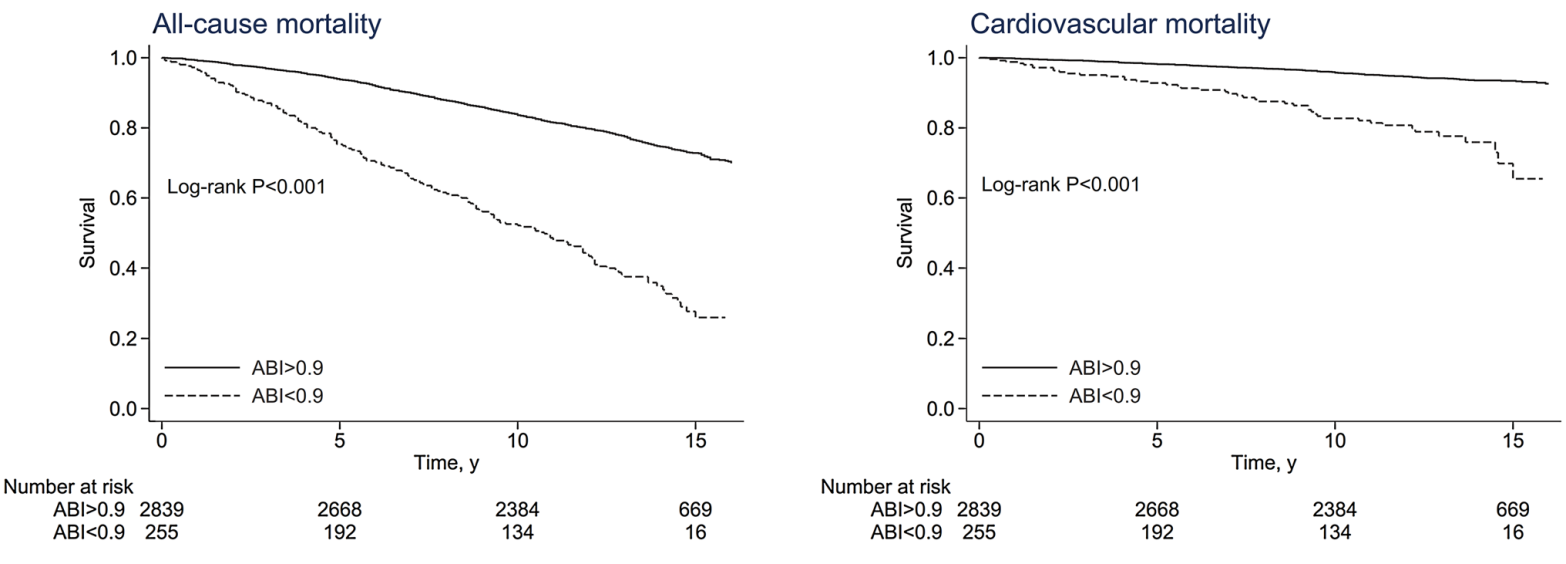
Kaplan–Meier (crude) survival curves, by peripheral arterial disease status, for all-cause mortality (left) and cardiovascular mortality (right). *ABI* ankle-brachial index

In crude Kaplan–Meier analyses, risks for both all-cause and cardiovascular mortality were significantly higher in participants with versus without PAD, as depicted in Fig. 2.

Multivariable-adjusted Cox proportional hazard models are shown in Table 3. After adjustment for age, race-ethnicity, education and BMI (Model 1), PAD was associated with a higher risk of all-cause (HR 2.2, 95% CI 1.7–2.8) and cardiovascular mortality (HR 3.3, 95% CI 2.1–5.3). The association remained significant after adjustment for additional risk factors including cigarette smoke, diabetes, blood pressure and prevalent CVD (Model 2). Sensitivity analyses performed in men and women separately confirmed the association between PAD and both all-cause and cardiovascular mortality in both sexes and in both multivariable-adjusted models. Results were also consistent when analysis were stratified according to FIB-4 levels (below or above 1.3), as shown in Supplementary Table 1. In this analysis, HRs were higher in participants without advanced liver fibrosis (i.e., FIB-4 < 1.3).

**Table 3:** Cox proportional hazard model evaluating the association between ankle-brachial index and all-cause and cardiovascular mortality in the studied population

	Hazard Ratio (95% CI)						
ABI	Entire cohort		Men			Women	
	Model 1^a^	Model 2^b^	Model 1^a^		Model 2^b^	Model 1^a^	Model 2^b^
All-cause mortality							
≥ 0.9	Reference	Reference	Reference		Reference	Reference	Reference
< 0.9	2.2 (1.7–2.8)	1.8 (1.4–2.4)	2.2 (1.6–3.0)		1.8 (1.3–2.5)	2.3 (1.5–3.6)	2.1 (1.4–3.1)
Cardiovascular mortality							
≥ 0.9	Reference	Reference	Reference		Reference	Reference	Reference
< 0.9	3.3 (2.1–5.3)	2.5 (1.5–4.3)	3.5 (1.9–6.5)		2.4 (1.3–4.6)	3.8 (1.6–8.9)	3.6 (1.4–8.8)

Results are presented as Hazard Ratios (95% Confidence Intervals). *P* < 0.01 for all analyses

*ABI* ankle-brachial index

^a^Model 1 adjusted for age, race, education and BMI

^b^Model 2 includes variables from Model 1 + diabetes, cigarette smoke, blood pressure, history of cardiovascular disease, HDL-cholesterol, statin use and chronic kidney disease

## Discussion

The present population-based cohort study provides the following main findings:

- The prevalence of PAD in patients with NAFLD from the general US population 40 years and older is substantial at approximately 6%.
- The ABI, a simple and non-invasive measure easily applicable to clinical practice, is associated with all-cause and cardiovascular mortality in these patients, in both sexes and even after adjustment for prevalent CVD and other risk factors.

While several studies estimated the prevalence of PAD in either the general population or patients with diabetes, few studies specifically focused on patients with NAFLD. In a recent study from China, comprising 1610 NAFLD patients older than 40 years, PAD (based on ABI) was present in 124 (7.7%), a proportion that is slightly higher compared with our estimate. Interestingly, it was associated with a higher risk of fibrosis progression (estimated through the NAFLD fibrosis score) [21].

As in previous studies, we show that patients with PAD are older, more frequently smokers, of non-Hispanic black ethnicity and affected by diabetes and hypertension [22]. The presence of these comorbidities might help identify subjects at particularly high risk, who might benefit most from an ABI evaluation. Sex also seems to play an important role, as patients with PAD were more frequently female compared with their counterparts. On this aspect, previous population studies have shown that women are at least as likely as men to have PAD across all age groups, but PAD prevalence seems to increase to a greater extent in females after the age of 70 [23, 24]. Independently of sex, our study shows that presence of PAD was associated with a doubled risk of all-cause mortality and a tripled risk of cardiovascular mortality during a mean follow-up time of 13 years, even after adjustment for several risk factors including a known history of CVD. While, to our knowledge, this is the first study evaluating the prognostic impact of PAD in a population of patients with NAFLD, similar results were obtained in several observational studies. In particular, a meta-analysis comprising 11 studies on a total of 44,590 subjects, showed that, even though significant heterogeneity existed across studies, a low ABI (< 0.9) was associated with an increased risk of all-cause mortality (RR 1.60, 95% CI 1.32–1.95) and cardiovascular mortality (pooled RR 1.96, 95% CI 1.46–2.64) [25]. Importantly, many patients with PAD are asymptomatic or show atypical leg pain, with only a minority presenting the classical intermittent claudication [26], but, when compared with symptomatic patients, their cardiovascular risk seems to be equally high [8]. Taken together, available evidence suggests to keep a high index of suspicion for this complex condition and that ABI measurement might be considered in patients at high cardiovascular risk, such as those with diabetes, metabolic syndrome and NAFLD, particularly if physical exam is consistent with the diagnosis [10].

The present study has several strengths. Being based on data obtained from NHANES survey cycles, it provides representative data that can be generalized to the entire US multiethnic adult population aged 40 years or older. Moreover, collection of both biochemical and risk factor data was performed in a standardized and homogenous fashion by trained personnel. Finally, we focused on hard clinical outcomes with very high degree of retention of participants, as data were based on the National Death Index.

On the same lines, several limitations should also be acknowledged. First, while FLI is a well-validated method for the diagnosis of steatosis, its performance is lower compared with more time-consuming and expansive techniques, such as magnetic resonance spectroscopy or liver biopsy. On the other hand, it is considered an acceptable alternative for the diagnosis of steatosis in large epidemiological studies by current clinical guidelines [7]. Second, all variables were measured at a single baseline examination and no data are available on development of PAD in the following years, leading to the possibility of misclassification. Third, data on certain variables, including smoking, alcohol use and prevalent CVD were based exclusively on self-report. Finally, despite adjustment for several risk factors for mortality, the possibility of residual confounding cannot be completely excluded.

In conclusion, we show that ~ 1 in 20 patients with NAFLD from the general US population has PAD, based on an ABI < 0.9. Moreover, PAD was significantly associated with an increased incidence of all-cause and cardiovascular mortality, in both men and women, and independently from cardiovascular risk factors. These findings suggest that measurement of ABI might be indicated in patients with NAFLD for cardiovascular risk assessment and prevention of CVD.

## Supplementary Information

Below is the link to the electronic supplementary material.

Supplementary file1 (DOCX 14 KB)

## Data Availability

The datasets are publicly available online at the NHANES website.
