# Supplementary material for: Peripheral artery disease and all-cause and cardiovascular mortality in patients with NAFLD
Source: J Endocrinol Invest. 2022 Apr 2;45(8):1547–53. doi: 10.1007/s40618-022-01792-9 (PMC9270293; doi:10.1007/s40618-022-01792-9)
Supplement: Supplementary file 1 — Supplementary file1 (DOCX 14 KB) [file 40618_2022_1792_MOESM1_ESM.docx]

**ONLINE SUPPLEMENT**

**Peripheral artery disease and all-cause and cardiovascular mortality in patients with NAFLD**

Stefano Ciardullo^1,2^, Eleonora Bianconi^1^, Rosa Cannistraci^1,2^, Paola Parmeggiani^1^, Enrico Maria Marone^3,4^, Gianluca Perseghin^1,2^

^1^Department of Medicine and Rehabilitation, Policlinico di Monza, Monza, Italy ^2^Department of Medicine and Surgery, University of Milano Bicocca, Milan, Italy, ^3^Vascular Surgery, Department of Clinical-Surgical, Diagnostic and Pediatric Sciences, University of Pavia. ^4^Department of Vascular Surgery, Policlinico di Monza, Monza, Italy

**Keywords**: NAFLD, ABI, mortality, CVD

**Running Title**: ABI and mortality in NAFLD

**Contact info**: Prof Gianluca Perseghin, Department of Medicine and Surgery, Università degli Studi di Milano Bicocca & Depart­ment of Medicine and Rehabilitation, Policlinico di Monza, Via Modigliani 10, 20900 Monza (MB), Italy, Email: gianluca.perseghin@policlinicodimonza.it or gianluca.perseghin@unimib.it

Phone +39 039 2810430

**Supplementary Table 1** Cox-proportional hazard model evaluating the association between ankle-brachial index and all-cause and cardiovascular mortality in the studied population.

| **Hazard ratio (95% CI)** | | |
| --- | --- | --- |
| **ABI** | **FIB-4 <1.3** | **FIB-4 ≥1.3** |
| All-cause mortality |  |  |
| ≥0.9 | Reference | Reference |
| <0.9 | 2.4 (1.5-3.6) | 1.5 (1.2-2.1) |
| Cardiovascular mortality |  |  |
| ≥0.9 | Reference | Reference |
| <0.9 | 4.5 (1.9-10.4) | 1.7 (1.0-2.9) |

Results are presented as Hazard Ratios (95% Confidence Intervals). P<0.01 for all analyses.

Results are adjusted for age, race, education, BMI, diabetes, cigarette smoke, blood pressure, history of cardiovascular disease, HDL-cholesterol, statin use and chronic kidney disease.
